# Clinical implication of ectopic liver lipid accumulation in renal cell carcinoma patients without visceral obesity

**DOI:** 10.1038/s41598-017-13209-2

**Published:** 2017-10-06

**Authors:** Daisuke Watanabe, Akio Horiguchi, Shinsuke Tasaki, Kenji Kuroda, Akinori Sato, Junichi Asakuma, Keiichi Ito, Tomohiko Asano, Hiroshi Shinmoto

**Affiliations:** 1 Department of Urology, National Defense Medical College, Tokorozawa-City, Saitama, Japan; 2 Department of Radiology, National Defense Medical College, Tokorozawa-City, Saitama, Japan

## Abstract

Fatty liver has emerged as a prognostic marker of cancer, so we investigated the impact of ectopic lipid accumulation in liver on the clinical outcome for patients with renal cell carcinoma (RCC). The records of 230 consecutive patients who had undergone surgery for RCC were reviewed, and liver lipid accumulation was estimated from the attenuation in unenhanced preoperative CT images. The median liver CT values of patients with G3 tumors was lower than that of patients with G1–2 tumors (*P* = 0.0116), that of patients with pT3–4 tumors was lower than that of patients with pT1–2 tumors (*P* = 0.0336), and that of patients with visceral obesity defined as a visceral fat area ≥ 100 cm^2^ was lower than that of patients without visceral obesity (*P* < 0.0001). In patients without visceral obesity the median liver CT values of patients with pT3–4 tumors was lower than that of patients with pT1–2 tumors (*P* = 0.0401), that of patients with metastasis was lower than that of patients without metastasis (*P* = 0.026), and fatty liver was associated with shorter overall survival (*P* = 0.0009). Ectopic lipid accumulation in liver thus seems to be a predictor of aggressive forms of RCC.

## Introduction

The obese population in the world continues to grow, and the diverse health problems associated with obesity have become a major social issue. It has been reported that obesity is associated with not only type 2 diabetes, dyslipidemia, and heart disease, but also with various types of cancers, including breast cancer, rectal cancer, and colon cancer^1^. However, there is great concern regarding the association between obesity and renal cell carcinoma (RCC). Although obesity defined by high BMI is well established as a risk factor for developing RCC, higher BMI is also associated with longer survival time, and this phenomenon has been called the obesity paradox in RCC^2^. To elucidate this paradox, studies considering obesity a change in body composition rather than a change in BMI have been made. Recently published reports investigating relations between obesity and clinicopathological parameters of RCC have used visceral and subcutaneous fat distribution as an index of obesity; however, there is no consensus yet on the linkage between RCC and body fat distribution^3–5^.

Excess lipid is stored not only as visceral and subcutaneous fat but also as ectopic fat. Excessive free fatty acids are accumulated in major adipose tissues in their storage form of triglyceride and also in organs other than adipose tissues (e.g., insulin target organs such as liver and skeletal muscles), where it is called ectopic fat^6^. Ectopic fat plays a role as an inflammation mediator, contributing to the induction of insulin resistance, and is believed to be linked with increased risks of lifestyle-related and cardiovascular diseases^7,8^. Particularly, with increased obese population not only in Europe and America but also in the Asia-Pacific Region, fat accumulation in the liver—specifically, the prevalence of increased fatty liver–is garnering attention as it is considered to be associated with increases in lifestyle-related diseases such as dyslipidemia, diabetes, and heart disease and with the progression of and survival rates for several kinds of cancer^9–11^. Considering the mechanism of liver lipid accumulation in which free fatty acids are directly transported to the liver from excessive visceral fat tissues through the portal vein and considering the relationship of liver lipid accumulation with both lifestyle-related diseases and cancer progression, it seems that liver lipid accumulation could represent excessive adiposity and that excessive lipid could have a great impact on the progression of RCC. In this study, we investigated the relevance of ectopic fat accumulation in the liver to the degree of malignancy and prognosis of RCC with a consideration given to visceral fat obesity.

## Results

### Relationship between visceral obesity and fatty liver

**Figure 1:**
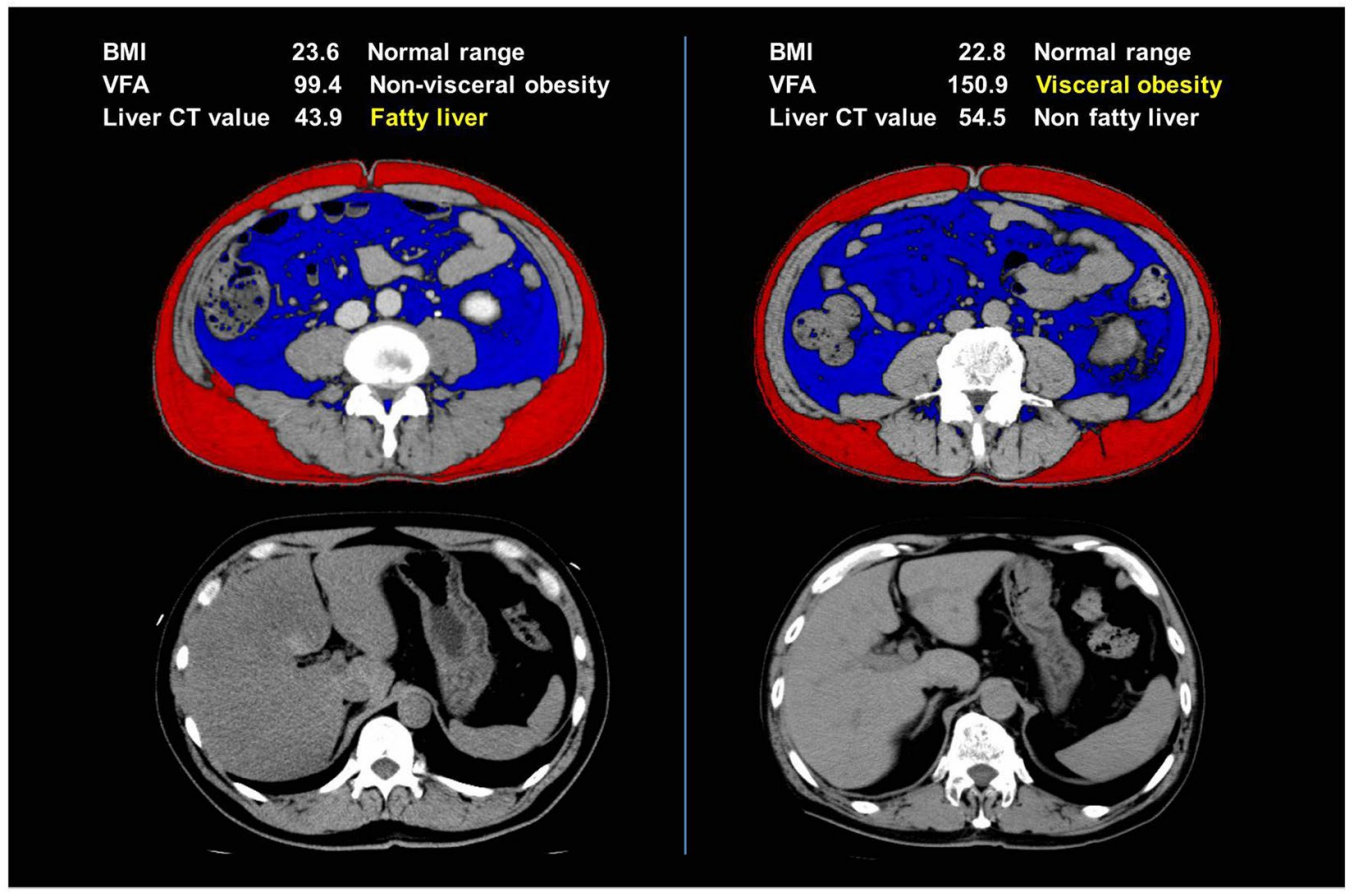
CT images of two representative RCC patients in the normal BMI range. BMI = body mass index (kg/m^2^), VFA = visceral fat area (cm^2^), Liver CT value = liver computed tomography value (Hounsfield units).

Clinicopathological characteristics of our cohort are listed in Table 1. The median age at surgery was 65 years (range 34–87) and median follow-up duration (from the date of operation to that of the last recorded follow-up) was 31.4 months (IQR 15.3–55.6), 14 patients died of cancer, and 6 patients died of other causes. BMI, VFA, and liver lipid accumulation differed widely. Even patients with a BMI in the normal range according to the World Health Organization (WHO) classification for Asian populations^12^ had excess fat: one patient had a fatty liver without visceral obesity, and another showed the inverse pattern (Fig. 1). Among the 230 patients in this cohort, visceral obesity was found in 129 (56.1%) and fatty liver was found in 40 (17.4%). Comparisons of clinicopathological features stratified by the presence or absence of visceral obesity are presented in Table 2. Patients with visceral obesity had a significantly higher BMI and lower liver CT value than those without visceral obesity (*P* < 0.0001 and *P* < 0.0001, respectively). Twenty-eight (21.7%) of the 129 patients with visceral obesity had fatty liver and this percentage is significantly higher than that in patients without visceral obesity (12 of 101, 11.9%, *P* = 0.048).

**Table 1:** Patient characteristics. ECOG-PS = Eastern Cooperative Oncology Group Performance Status; IQR = interquartile range.

Characteristics		Overall patients
Total	No. (%)	230 (100)
Gender	No. (%)	
Male		177 (77.0)
Female		53 (23.0)
Age	Median (range)	65 (34–87)
Follow-up months	Median (IQR)	31.4 (15.3–55.6)
ECOG-PS	No. (%)	
PS0–1		224 (97.3)
PS2–4		6 (2.6)
Visceral obesity	No. (%)	
negative		101 (43.9)
positive		129 (56.1)
Fatty liver	No. (%)	
negative		190 (82.6)
positive		40 (17.4)
Grade	No. (%)	
G1		17 (7.4)
G2		107 (46.5)
G3		106 (46.1)
Venous invasion	No. (%)	
negative		136 (59.1)
positive		94 (40.9)
Growth pattern	No. (%)	
Expansive		175 (76.1)
Infiltrative		55 (23.9)
Pathological T stage	No. (%)	
T1–2		192 (83.5)
T3–4		38 (16.5)
Clinical N/M stage	No. (%)	
N0M0		208 (90.4)
N1–2 and/or M1		22 (9.6)

**Table 2:** Characteristics of patients with visceral obesity and non-visceral obesity. BMI = body mass index; CT = computed tomography; IQR = interquartile range; VFA = visceral fat area; *P*-value < 0.05 marked in bold font shows statistical significance.

Characteristics	Visceral obesity			Non-visceral obesity		*P*
	VFA ≥ 100 cm^2^			VFA < 100 cm^2^		
Patients	No. (%)	129	(56.1)	101	(43.9)	
Median age	(IQR)	65	(57–73)	65	(59–73)	0.525
Median follow-up months	(IQR)	30.5	(15.3–48.6)	34.8	(15.3–66.7)	0.103
Median BMI	(IQR)	24.7	(22.7–26.7)	20.7	(19.5–22.5)	**<0.0001**
Median liver CT value	(IQR)	56.5	(51.7–61.8)	60.0	(56.8–64.8)	**<0.0001**
Fatty liver	No. (%)	28	(21.7)	12	(11.9)	**0.048**
Grade	No. (%)					0.4931
G1		10	(7.8)	7	(6.9)	
G2		64	(49.6)	43	(42.6)	
G3		55	(42.6)	51	(50.5)	
Venous invasion	No. (%)					0.462
positive		50	(38.8)	44	(43.6)	
negative		79	(61.2)	57	(56.4)	
Growth pattern	No. (%)					0.232
Expansive		102	(79.1)	73	(72.3)	
Infiltrative		27	(20.9)	28	(27.7)	
Pathological T stage	No. (%)					0.058
T1–2		113	(87.6)	79	(78.2)	
T3–4		16	(12.4)	22	(21.8)	
Clinical N/M stage	No. (%)					0.051
N0M0		121	(93.8)	87	(86.1)	
N1–2 and/or M1		8	(6.2)	14	(13.9)	

### Influence of liver lipid accumulation on RCC

**Figure 2:**
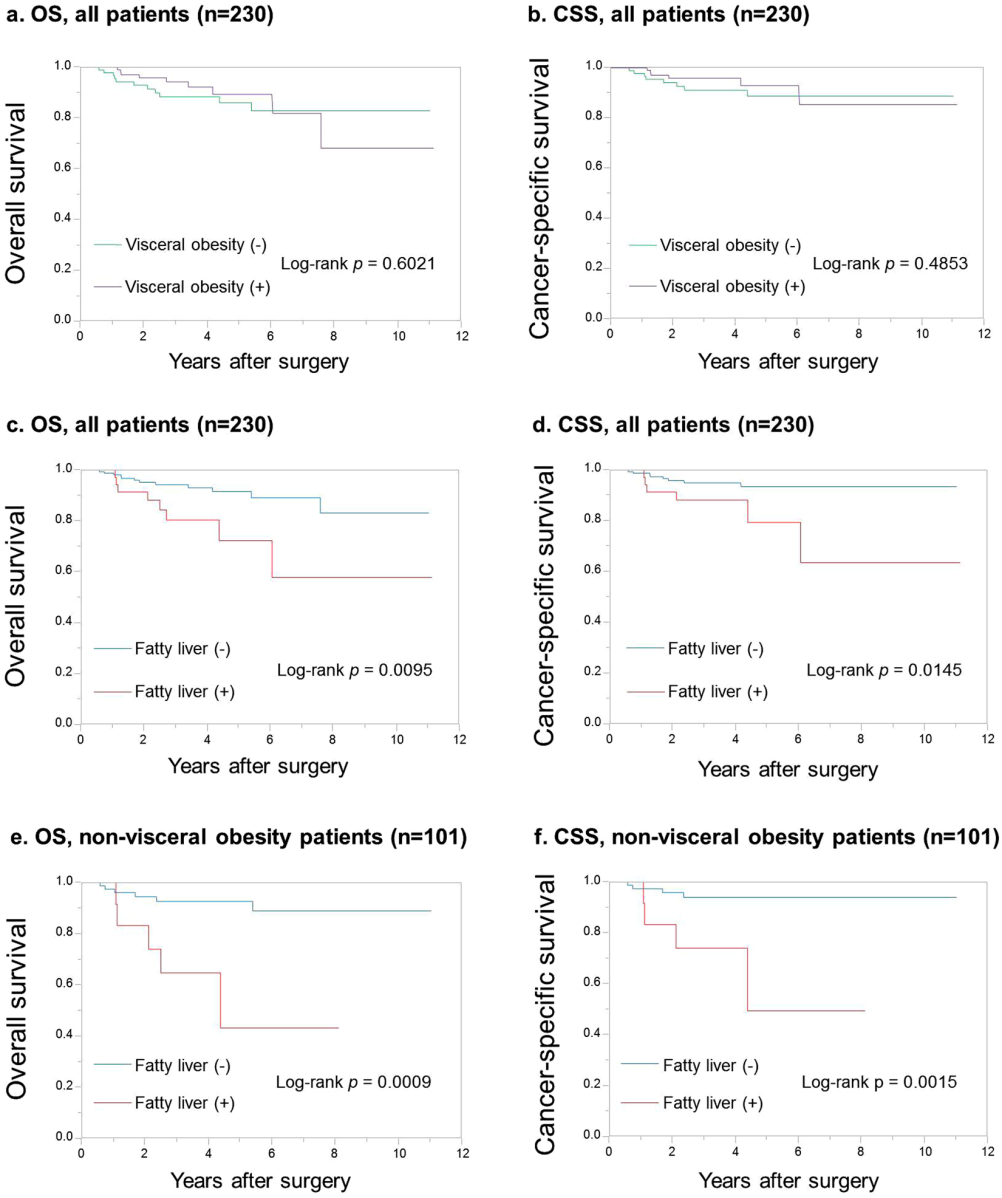
Kaplan-Meier curves for overall survival (OS) and cancer-specific survival (CSS) stratified by visceral obesity and fatty liver. OS (**a**) and CSS (**b**) in all patients according to visceral obesity. OS (**c**) and CSS (**d**) in all patients according to fatty liver. OS (**e**) and CSS (**f**) in patients without visceral obesity according to fatty liver.

Presence of visceral obesity was not associated with any clinicopathological parameters including pathological stage, tumor grade, venous invasion, or survival (Table 2, Fig. 2a,b). On the other hand, the median liver CT values of patients with high-grade tumors (G3) was lower than that of patients with low-grade tumors (G1–2) (Table 3, *P* = 0.0116), that of patients with pT3–4 tumors was lower than that of patients with pT1–2 tumors (Table 3, *P* = 0.0336), and the pathological T stage of patients with fatty liver was higher than that of patients without fatty liver (Table 4, *P* = 0.0397). The OS and CSS of patients with fatty liver were shorter than those of patients without fatty liver (Fig. 2c,d, *P* = 0.0095 and *P* = 0.0145, respectively).

**Table 3:** Association between liver CT value and clinicopathological parameters in overall patients and in patients without visceral obesity. BMI = body mass index; CT = computed tomography; IQR = interquartile range; LN = lymph node; *P*-value < 0.05 marked in bold font shows statistical significance.

Parameter		Overall patients				Patients without visceral obesity			
		N	Liver CT value	IQR	*P*	N	Liver CT value	IQR	*P*
Grade	G1–2	124	59.3	54.1–64.5	**0.0116**	50	62.6	58.2–65.3	0.0632
	G3	106	57.1	51.2–61.9		51	59.3	54.9–63.5	
Venous invasion	negative	136	58.7	54.1–63.3	0.3434	57	59.8	57.9–63.8	0.959
	positive	94	57.5	52.2–63.1		44	60.4	55.0–65.3	
Growth pattern	Expansive	175	58.7	54.0–63.7	0.1801	73	60.7	57.7–65.2	0.1069
	Infiltrative	55	57.6	51.4–61.9		28	59.2	52.2–63.1	
Pathological T stage	T1–2	192	58.7	54.1–63.4	**0.0336**	79	60.8	58.1–65.0	**0.0401**
	T3–4	38	56.6	49.7–60.7		22	58.0	49.9–63.7	
LN and/or distant metastasis	N0M0	208	58.6	53.6–63.4	0.1615	87	60.8	57.7–65.0	**0.026**
	N1–2 and/or M1	22	57.3	49.7–60.3		14	57.3	49.7–60.3

**Table 4:** Clinicopathological characteristics of patients with fatty liver and patients without fatty liver. *P*-value < 0.05 marked in bold font shows statistical significance.

Parameter		Overall patients			Patients without visceral obesity		
		Fatty liver	Non-fatty liver	*P*	Fatty liver	Non-fatty liver	*P*
Grade	No. (%)			0.1503			0.1694
G1		2 (5.0)	15 (7.9)		0 (0.0)	7 (7.9)	
G2		14 (35.0)	93 (49.0)		3 (25.0)	40 (44.9)	
G3		24 (60.0)	82 (43.1)		9 (75.0)	42 (47.2)	
Venous invasion	No. (%)			0.0997			0.2717
negative		19 (47.5)	117 (61.6)		5 (41.7)	52 (58.4)	
positive		21 (52.5)	73 (38.4)		7 (58.3)	37 (41.6)	
Growth pattern	No. (%)			0.1613			0.0663
Expansive		27 (67.5)	148 (77.9)		6 (50.0)	67 (75.3)	
Infiltrative		13 (32.5)	42 (22.1)		6 (50.0)	22 (24.7)	
Pathological T stage	No. (%)			**0.0397**			**0.0116**
T1–2		29 (72.5)	163 (85.8)		6 (50.0)	73 (82.0)	
T3–4		11 (27.5)	27 (14.2)		6 (50.0)	16 (18.0)	
Clinical N/M stage	No. (%)			0.0605			**0.003**
N0M0		33 (82.5)	175 (92.1)		7 (58.3)	80 (89.9)	
N1–2 and/or M1		7 (17.5)	15 (7.9)		5 (41.7)	9 (10.1)	

### Influence of liver lipid accumulation in non-visceral fat obesity on RCC

To examine the impact of liver lipid accumulation on clinicopathological parameters and clinical outcome in patients with RCC, we further analyzed its impact stratified by the presence of visceral obesity. In patients without visceral obesity the median liver CT values of patients with pT3–4 tumors was lower than that of patients with pT1–2 tumors (Table 3, *P* = 0.0401), that of patients with lymph node (LN) metastasis and/or distant metastasis at the time of operation was lower than that of patients without any metastasis (Table 3, *P* = 0.026), the pathological T stage of patients with fatty liver was higher than that of patients without fatty liver (Table 4, *P* = 0.0116), and patients with fatty liver were more positive for LN metastasis and/or distant metastasis than patients without fatty liver (Table 4, *P* = 0.003). The OS and CSS of patients with fatty liver were shorter than those of patients without fatty liver (Fig. 2e,f, *P* = 0.0009 and *P* = 0.0015, respectively).

## Discussion

It is well known that obesity is generally associated with increased risk of RCC^1,13^. The field of obesity has recently moved to the evaluation of body fat distribution measuring by CT. Visceral adiposity, or a large VFA, has been widely used as an index of obesity from the aspect of metabolic activity and sensitivity to lipolysis and insulin-resistance in adipocytes^14^. Different associations between VFA and clinicopathological parameters and clinical outcome of RCC have been reported. Although some reports suggest the associations between higher VFA and better clinicopathological outcome and better survival rates^5,15,16^, one suggested an association between higher VFA and higher tumor aggressiveness^4^, one suggested no association between higher VFA and higher tumor aggressiveness^17^, and one recently a U-shaped association between VFA/total adipose area ratio and recurrence risk^3^. Currently, there is no consensus on the clinical significance of visceral obesity with regard to the pathological parameters and outcome of patients with RCC.

The liver has recently been found to be an organ in which ectopic accumulation of excessive fat occurs. Though the mechanism of lipid accumulation in the liver is unclear, two theories are generally accepted: one is a “spillover concept” in which free fatty acids from excessive visceral fat tissues are transported directly to the liver through the portal vein and accumulated as triglyceride, and the other is “changes in hepatocyte lipid metabolism” in which insulin resistance associated with obesity induces fatty acid re-esterification in hepatocyte, resulting in increases in de novo lipogenesis^18^. In other words, the former theory reflects the exceeded threshold of accumulated visceral fat, and the latter theory reflects the exacerbation of insulin resistance. Excess adiposity is in the state of having a lot of triglyceride overloaded and hypertrophied adipocytes, which are known to lead more secretion of tumor necrosis factor α (TNF-α) and leptin and less secretion of adiponectin^19^. It is generally indicated that TNF-α promotes progression of RCC by enhancing tumor invasion and epithelial-mesenchymal transition and that lower serum adiponectin levels and higher serum leptin levels are associated with higher aggressiveness of RCC^20–23^. In addition, increased serum insulin-like growth factors and insulin levels induced by insulin resistance are generally thought to be associated with tumor development and progression of RCC^24^.

In Asia there are many patients with fatty liver despite the fact that they are not visceral obese individuals. According to previous epidemiological case reports, fatty liver in non-obese patients in Asia is not negligible and the ratio of non-alcoholic fatty liver disease (NAFLD) is approximately 10%^25,26^. In our cohort, of the 40 patients with fatty liver, 28 showed visceral obesity and 12 did not (Table 2). In patients without visceral obesity, liver lipid accumulation is significantly associated with high pathological T stage and LN/distant metastasis (Table 3, *P* = 0.0401 and *P* = 0.026, respectively). These clinicopathological significances in fatty liver patients without visceral obesity suggest that the pathogenesis of non-spillover type fatty liver (e.g.; existence of insulin resistance) is involved in the aggressiveness and proliferation of RCC. Although there are various possible reasons for people develop a fatty liver develop despite being non-obese, such as involvement of a genetic factor (i.e., *PNPLA3* expression)^27^, it is generally considered non-obese subjects with fatty liver are in the state of insulin resistance and other metabolic disorder compared with non-obese subjects without fatty liver^28^, and it is also conceivable that insulin resistance may play an important role in the development and progression of RCC in these subjects.

Our study is the first showing that liver lipid accumulation defined by CT values is correlated with specific clinicopathological factors and decreases in OS rate and CSS rate, and it also the first study considering patients without visceral obesity. Although it has been recognized that fatty liver leads to chronic liver disease and hepatocellular carcinoma, there are few reports regarding the association between fatty liver and cancer in other organs. A Danish cohort study found alcoholic fatty liver to increase the risk of lung cancer and breast cancer and found NAFLD to increase the risk of pancreatic cancer and kidney cancer, and it is the only previous report mentioning fatty liver in the context of kidney cancer risk^11^. In clinical practice, abdominal ultrasound and CT value measurement are becoming widely used to detect fatty liver. There is a high probability that a preoperative CT scan is performed for the diagnosis of RCC. In addition, periodic CT scans after surgery for RCC are needed to confirm the absence of recurrence. CT values obtained from an unenhanced liver CT scan, unlike visceral fat areas measured using specialized software, are markers that can be easily measured by those who are not radiation imaging experts. They could be RCC predictors useful in terms of preoperative detecting simplicity for urologists and could be used for stratifying follow-up protocols.

There were several limitations in this study. Firstly, the subjects of this study were all Japanese, but the cutoff value of each parameter in defining obesity would be different for each race and gender. The definition of visceral obesity in Japanese is VFA ≥100 cm^2^ regardless of gender, and the BMI for WHO classification of obesity in Asian populations is ≥25 regardless of gender. Therefore the results of the present study may be applicable only to Asians. Secondly, elements such as history of drinking, drug use, and viral hepatitis infection and their effects on liver adiposity were not taken into consideration in the present study, and therefore it is possible that impact of these elements on malignancy and prognosis of RCC may be included. Thirdly, this was a retrospective study conducted at our single facility; therefore, there may be a bias in subject patient selection. Despite these limitations, findings of the present study confirmed the significant impact of ectopic lipid accumulation in the liver on clinicopathological parameters and clinical outcome of RCC. Further studies on this subject are needed. Ectopic lipid accumulation in the liver can be easily evaluated with preoperative CT images and can be a useful predictive factor for postoperative prognosis.

## Methods

### Study Cohort

A total of 230 patients (177 male and 53 female) who underwent partial or radical nephrectomy for RCC at National Defense Medical College Hospital, Saitama, Japan, from July 2003 to January 2014 were retrospectively reviewed. The protocol and informed consent for the retrospective research were approved by the Ethics Committee of National Defense Medical College. All methods were approved by the Ethics Committee of National Defense Medical College and were carried out in accordance with the approved guidelines. All tumor tissues were evaluated for pathological staging and histological grading according to the 7th TNM classification of the AJCC (American Joint Committiee on Cancer) and the UICC (Union International Centre le Cancer)^29^. We assessed the age, gender, Eastern Cooperative Oncology Group Performance Status (ECOG-PS) scale, the presence or absence of visceral obesity and fatty liver, body mass index (BMI), regional lymph node involvement, presence of distant metastasis, and various pathological parameters.

### Image Analysis

The visceral fat area (VFA) in the preoperative unenhanced computed tomography (CT) images at the umbilical level was estimated using imaging software (EV Insite, PSP Corporation, Tokyo, Japan). Because all subjects were Japanese, visceral obesity was defined by a VFA ≥ 100 cm^2^ according to the criteria of JASSO (Japan Society for the Study of Obesity)^30^. CT has been widely used to evaluate the liver lipid accumulation because of its high sensitivity and specificity^31^, and in this study CT values of liver were estimated in the preoperative unenhanced CT images. All evaluated unenhanced CT images are set with the same Window Width (fixed at 250) and same Window Level (fixed at 50). In order to capture the entire liver as much as possible, three different regions of interest (ROI) without vascular area including the left hepatic lobe (S3), the anterior segment of right hepatic lobe (S6), and the posterior segment of right lobe (S8) were selected, and the mean CT values of these ROI averaged to estimate the liver CT value. As the normal range of liver CT value at unenhanced CT images is generally 50–65 Hounsfield units (HU)^32^, a fatty liver was defined in this study as one with a mean CT value < 50 HU.

### Statistical Analysis

Within each group, the Wilcoxon rank sum test was used for comparisons of continuous variables and the chi-squared test was used for comparisons of categorical variables. Overall survival (OS) and cancer-specific survival (CSS) rates were compared using the Kaplan-Meier method with the log-rank test. *P* values less than 0.05 were considered to indicate statistical significance. All statistical analyses were performed using JMP® 10 (SAS Institute Inc., Cary, NC, USA).
